# Prevalence of Familial Hypercholesterolemia in Pakistan: A Pooled Analysis of 1.5 Million Individuals and Comparison with Other Countries of the Region

**DOI:** 10.5334/gh.1413

**Published:** 2025-03-10

**Authors:** Madeeha Khan, Quratul Ain, Jaka Sikonja, Hijab Batool, Muhammad Qasim Hayat, Mohammad Iqbal Khan, Urh Groselj, Fouzia Sadiq

**Affiliations:** 1 Atta ur Rehman School of Applied Biosciences, National University of Sciences and Technology, Islamabad, Pakistan; 2 Shifa Tameer-e-Millat University, Islamabad, Pakistan; 3 Translational Genomics Laboratory, Department of Biosciences, COMSATS University Islamabad, Pakistan; 4 Department of Endocrinology, Diabetes, and Metabolic Diseases, University Children’s Hospital, University Medical Centre Ljubljana, Bohoriceva ulica 20, Ljubljana, Slovenia; 5 Faculty of Medicine, University of Ljubljana, Vrazov trg 2, Ljubljana, Slovenia; 6 Chemical Pathology, Chughtai Institute of Pathology, Lahore, Pakistan; 7 Department of Vascular Surgery, Shifa Tameer-e-Millat University, Shifa International Hospital Islamabad, Pakistan

**Keywords:** Familial hypercholesterolemia, Prevalence, Cardiovascular disease, Pakistan, South Asia, Southeast Asia, Screening

## Abstract

**Background:**

Familial hypercholesterolemia (FH) is an inherited disorder that causes elevated LDL-C levels leading to premature cardiovascular disease but remains underdiagnosed. This study aims to determine the prevalence of FH in Pakistan using data from multiple laboratory networks and compare it with other counties of the region.

**Methods:**

The study analyzed lipid profile data from two large laboratory networks in Pakistan, applying Make Early Diagnosis to Prevent Early Death (MEDPED) LDL-C criteria for the general population to identify FH cases. A pooled prevalence estimate of prevalence of FH in Pakistan was calculated by combining the data of studies reporting prevalence in Pakistan. A systematic review was conducted to assess FH prevalence in South and Southeast Asian countries.

**Results:**

Analysis of 545,087 individuals (Median age 45 years, 58.2% males) identified 2,911 FH cases [0.55%, 95% confidence interval (CI): 0.53–0.57%), equivalent to a prevalence of 1:182. Pooled analysis with a previous Pakistani study, totaling 1,533,393 subjects, estimated the overall FH prevalence in Pakistan at 1:273 (95% CI: 0.21–0.64%). Prevalence decreased with age, being highest in the <20 years group (1:29), while no gender differences were observed. A systematic review of South and Southeast Asian countries revealed limited data, with FH prevalence estimates missing for majority of the countries of the region.

**Conclusion:**

This study provides an updated estimate of FH prevalence in Pakistan and highlights the scarcity of data in South and Southeast Asia.

## Introduction

Familial hypercholesterolemia (FH) is an autosomal codominant disease characterized by the reduced clearance of low-density lipoprotein cholesterol (LDL-C) particles from the blood due to pathological variants in the genes for low-density lipoprotein receptor (*LDLR*), apolipoprotein B (*APOB*) or proprotein convertase subtilisin/kexin type 9 *(PCSK9*) (1). This can result in premature cardiovascular morbidity and mortality due to atherosclerotic cardiovascular disease (ASCVD) that accounts for more than 19 million deaths annually (2,3).

Familial hypercholesterolemia remains highly underdiagnosed and undertreated throughout the world despite being recognized as a global health priority by the World Health Organization (4,5). Early diagnosis and screening of FH can significantly lower the burden of cardiovascular events that may develop due to missed diagnosis. Identification of prevalence estimates could be beneficial in highlighting the underdiagnosis of the disease and could be instrumental in designing strategies aimed at screening patients with FH (5,6).

Previously, the global prevalence of FH was estimated at around 1:500 (7,8) however, recent meta-analyses indicate the global prevalence of FH to be 1:313, however the prevalence estimates are missing for 90% of the countries (6). The early diagnosis of FH, followed by required treatment, is essential to prevent or at least delay the onset of cardiovascular events (9). Lack of financial resources and no consolidated guidelines for screening of FH remains a major challenge in Pakistan (10). Previously the prevalence of FH in Pakistan was estimated to be 1:409 (11).

While a previous study conducted in Pakistan identified FH prevalence using data from one laboratory network primarily in the Sindh province, the present study provides an expanded overview by calculating the prevalence of FH from two other large country-wide laboratory networks, with significant representation from Punjab and the Federal Capital Territory. This study also includes prevalence estimates for the pediatric population. Moreover, we provide pooled prevalence estimates of FH in Pakistan, combining our data with the previous study. Additionally, we conducted a systematic review to analyze the prevalence of FH in other countries of the South and Southeast Asia region, addressing the gap in comprehensive regional data.

## Methodology

### Primary data

#### Study Data

Anonymized retrospective data for lipid profile between March 2019 to March 2024 was obtained from databases of a network of diagnostic centers and collection points of Shifa International Hospital, Islamabad and Chughtai Laboratories, Lahore, Pakistan. In the case of repeated measurements of a single individual, only the value of the first test was included in the analysis, hence 159,504 repeated tests were removed. Apart from this, data of individuals >70 years (n = 9,934), missing city of residence (n = 1,879), and missing LDL-C measurements (n = 28,873) were excluded from the study (Supplementary Figure 1). The LDL-C was measured directly by using homogenous enzymatic methods, Cobas 8000 c502 module by Roche Diagnostics, USA, at Shifa International Hospital, and the Abbott Alinity ci analyzer at Chughtai Laboratories. Institutional Review Board and Ethics Committee (IRB&EC), Shifa Tameer-e-Millat University, Islamabad, Pakistan approved this study (IRB number 0323-22).

#### FH criteria

Familial hypercholesterolemia was defined by the MEDPED LDL-C criteria (12). Since the information about family history was not known, FH was defined based on LDL-C cutoff values 200 mg/dL for subjects younger than 20 years, 220 mg/dL for those from 20–29 years, 240 mg/dL for those from 30–39 years, and 260 mg/dL for subjects 40 years or older as reported by (12). Since the data was derived from databases of the diagnostic centers, the data on lipid lowering therapy was not available so LDL-C was not corrected.

#### Systematic review

A comprehensive systematic review until May 2024 was conducted to identify studies reporting prevalence of FH in South and Southeast Asian countries on four databases: PubMed, Web of Science, Scopus, and Google Scholar. The search was conducted using a combination of keywords ‘familial hypercholesterolemia’, ‘screening’ or ‘prevalence’ or ‘incidence’ or ‘epidemiology’ or ‘frequency’ and ‘South Asia’ or ‘Southeast Asia’ or specific countries within these regions (Supplementary materials section 1.1). Studies were included if they were conducted in South and Southeast Asian countries; were published in English; and had full-text availability or sufficient information in the abstract. Studies were excluded if they provided insufficient information on FH criteria used; or focused on specific subgroups, such as patients with premature coronary artery disease or familial combined hyperlipidemia. Moreover, all studies were included regardless of their publication date. The systematic review was conducted, according to the Preferred Reporting Items for Systematic Reviews and Meta-Analyses (PRISMA) guidelines.

### Study selection and data extraction

Two independent reviewers screened titles and abstracts of all identified studies. Full texts of potentially eligible studies were then assessed against the inclusion and exclusion criteria. Any disagreements were resolved through discussion or consultation with a third reviewer. Data were extracted using a standardized form, including study characteristics (author, year, country, setting, sample size, FH diagnostic criteria used, and prevalence or frequency of FH) (Supplementary Table 1).

#### Quality assessment

The quality of included studies was assessed independently by two authors using Joanna Briggs Institute (JBI) Prevalence Critical Appraisal Tool, which consists of nine items evaluating key methodological aspects such as sampling methods, sample size, data collection procedures, measurement validity, and statistical analysis. Each item, if fulfilled, generates one point, resulting in a score range from 0 to 9. Discrepancies of more than two points between the authors’ scores were resolved through consensus. The average of the two scores was used to categorize study quality for subgroup analyses: low quality (0–3 points), moderate quality (4–6 points), and high quality (7–9 points).

### Statistical analysis

#### Calculation of prevalence from primary data

The continuous variables such as age and LDL-C levels were reported as median and interquartile ranges (IQR), while categorical variables were presented as frequencies and percentages. A binary variable indicating FH status was then reported as a categorical variable with frequencies and percentages. Confidence intervals (CIs) of 95% for the prevalence estimate were determined using the Wilson score method. The comparisons between the groups were conducted by chi square test. For all comparisons p value of <0.05 was considered significant. The analysis was conducted in SPSS version 26 and R version 4.4.1.

#### Pooled meta-analysis for prevalence in Pakistan

Pooled prevalence estimates for prevalence in Pakistan was conducted for the data used in this study with other published studies from Pakistan. Heterogeneity across studies was assessed using the I² statistic. A high level of heterogeneity (I² > 75%) is often expected in studies of this nature (6). Therefore, to account for the high heterogeneity, a random-effects model was employed using the Generalized Linear Mixed Models (GLMM) method. This method is effective in handling high variability and complex data structures by accounting for both within-study and between-study variance. A subgroup analysis of age and gender was conducted, and the estimates were plotted on a forest plot. The meta-analysis was performed using the metaprop function from the meta package in R version 4.4.1.

#### Mapping

The visit locations of individuals were recorded at the district level and subsequently traced to their respective provinces and administrative units. Spatial coordinates for these locations were extracted using Google Maps. For map creation, shapefiles for Pakistan and Southeast Asia were obtained from https://earthworks.stanford.edu/. The maps were then developed using QGIS version 3.36.0.

## Results

### Primary data

#### General characteristics of the cohort

A total of 545,087 individuals were included in the final analysis. The characteristics of the included participants are given in Table 1. The median (IQR) age was 45 (36–35) years and majority (n = 317,446, 58.2%) of the individuals were male. The overall median (IQR) LDL-C levels were 124 (97–151) mg/dL. Most of the individuals belonged to the province of Punjab (n = 477,261, 87.5%) followed by Sindh (n = 24,104, 4.4%), Khyber Pakhtunkhwa (n = 23,140, 4.2%), and Islamabad Capital Territory (ICT, n = 12,347, 2.2%).

**Table 1:** Demographic characteristics of the study population (n = 545,087).


CHARACTERISTICS	STUDY DATA

Total participants, N	545,087

Median age (IQR) years	45 (36–55)

**Age distribution, n (%)**	

<20 years	8,087 (1.48)

20–29 years	4,6504 (8.53)

30–39 years	124,168 (22.77)

>40 years	366,328 (67.20)

**Gender, n (%)**	

Male	317,446 (58.23)

Female	227,636 (41.76)


#### FH prevalence

After applying MEDPED criteria, 2,911 [0.55% (95% CI = 0.53–0.57%)] individuals fulfilled the criteria for FH that is estimated to be 1:182 individuals. The prevalence was highest in individuals [4.81% (95% CI = 4.35–5.30%), 1:29] aged <20 years, followed by those aged between 20–29 years [1.60% (95% CI = 1.48–1.71%), 1:163], 30–39 years [0.70% (95% CI = 0.65–0.74%), 1:144] and >40 years [0.27% (95% CI = 0.26–0.29%), 1:368] (p < 0.01). In pediatric population, for children less than 10 years of age, the prevalence was even higher [11.75% (95% CI = 10.07–13.60%), 1:8], while those aged between 10–18 years had a prevalence of 3.62% (95% CI = 3.13–4.17%, 1:28). The prevalence was slightly higher in males [0.56% (95% CI = 0.53–0.58%), 1:180] compared to females [0.54% (95% CI = 0.51–0.57%), 1:185], however the difference was not statistically significant (p = 0.66) (Table 2).

**Table 2:** Familial Hypercholesterolemia Prevalence by Age and Gender in the primary data (n = 545,087).


CATEGORY	FH CASES	N	PREVALENCE, % (95% CI)	PREVALENCE PER 1000	PROPORTION	P VALUE

Overall	2,991	545,087	0.55 (0.53–0.57)	5.5	1:182	

**Gender**						

Male	1,766	317,446	0.56 (0.53–0.58)	5.6	1:180	0.66

Female	1,225	227,641	0.54 (0.51–0.57)	5.4	1:185	

**Age groups**						

<20 years	389	8,087	4.81 (4.35–5.30)	48.1	1:21	<0.01

20–29 years	742	46,504	1.60 (1.48–1.71)	16.0	1:63	

30–39 years	864	124,168	0.70 (0.65–0.74)	7.0	1:144	

>40 years	996	366,328	0.27 (0.26–0.29)	2.7	1:368	


**Figure 1:**
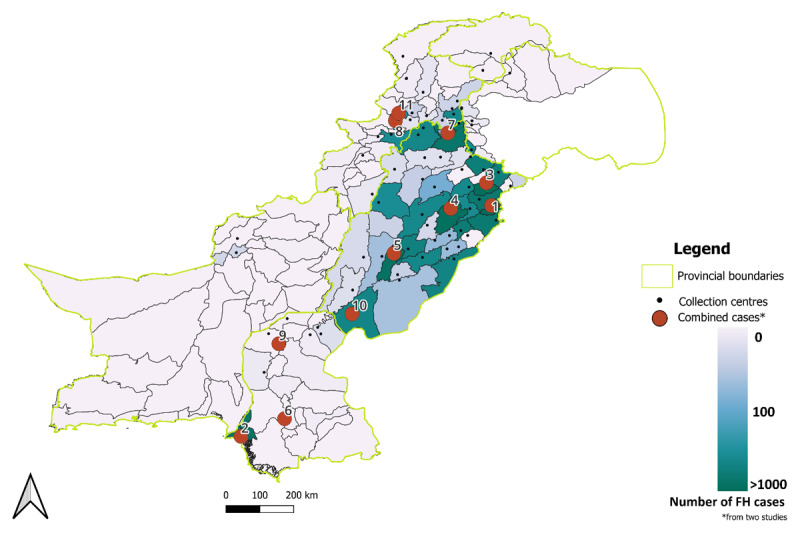
**Map of Pakistan with district wise prevalence of FH**. The black dots represent districts from which data was received. The red dots represent districts in which combined prevalence (present study and Farhad et al, 2023) was estimated. 1. Lahore (FH cases = 1236), 2. Karachi (FH cases = 1069), 3. Gujranwala (FH cases = 233), 4. Faisalabad (FH cases = 216), 5. Multan (FH cases = 170), 6. Hyderabad (FH cases = 159), 7. Rawalpindi (FH cases = 100), 8. Peshawar (FH cases = 86), 9. Larkana (FH cases = 69), 10. Rahim Yar Khan (FH cases = 62), 11. Sukkar (FH cases = 61).

A greater proportion of individuals with FH were from the Punjab province (n = 2,594, 86.7%) followed by Khyber Pukhtunkhwa (n = 179, 6.0%), Sindh (n = 128, 4.3%), ICT (n = 49, 1.6%), Azad Jammu and Kashmir (n = 23, 0.7%), and Balochistan (n = 18, 0.6%) (Figure 1). In Punjab and overall, the highest proportion of cases were reported from Lahore (n = 1101, 37%) followed by Gujranwala (n = 200, 6.7%), Faisalabad (n = 134, 4.5%), and Multan (n = 117, 3.9%) (Figure 1).

### Systematic Review

#### Study selection

The systematic review identified a total of 1,290 studies through database searches in PubMed, Web of Science, Scopus, and Google Scholar. After removing duplicates, 1,022 studies remained for title and abstract screening. Following this initial screening, seven studies were deemed potentially eligible and underwent full-text review. Ultimately, six studies met the inclusion criteria and were included in the final analysis. The PRISMA flow diagram detailing the study selection process is presented in Supplementary Figure 2.

#### Quality assessment

Based on the scores for quality assessment, two studies qualified as moderate quality, while the other four studies were high quality (Supplementary table 1). The primary areas of methodological weakness included sampling methods and measurement validity.

#### Pooled prevalence estimates for Pakistan

**Figure 2:**
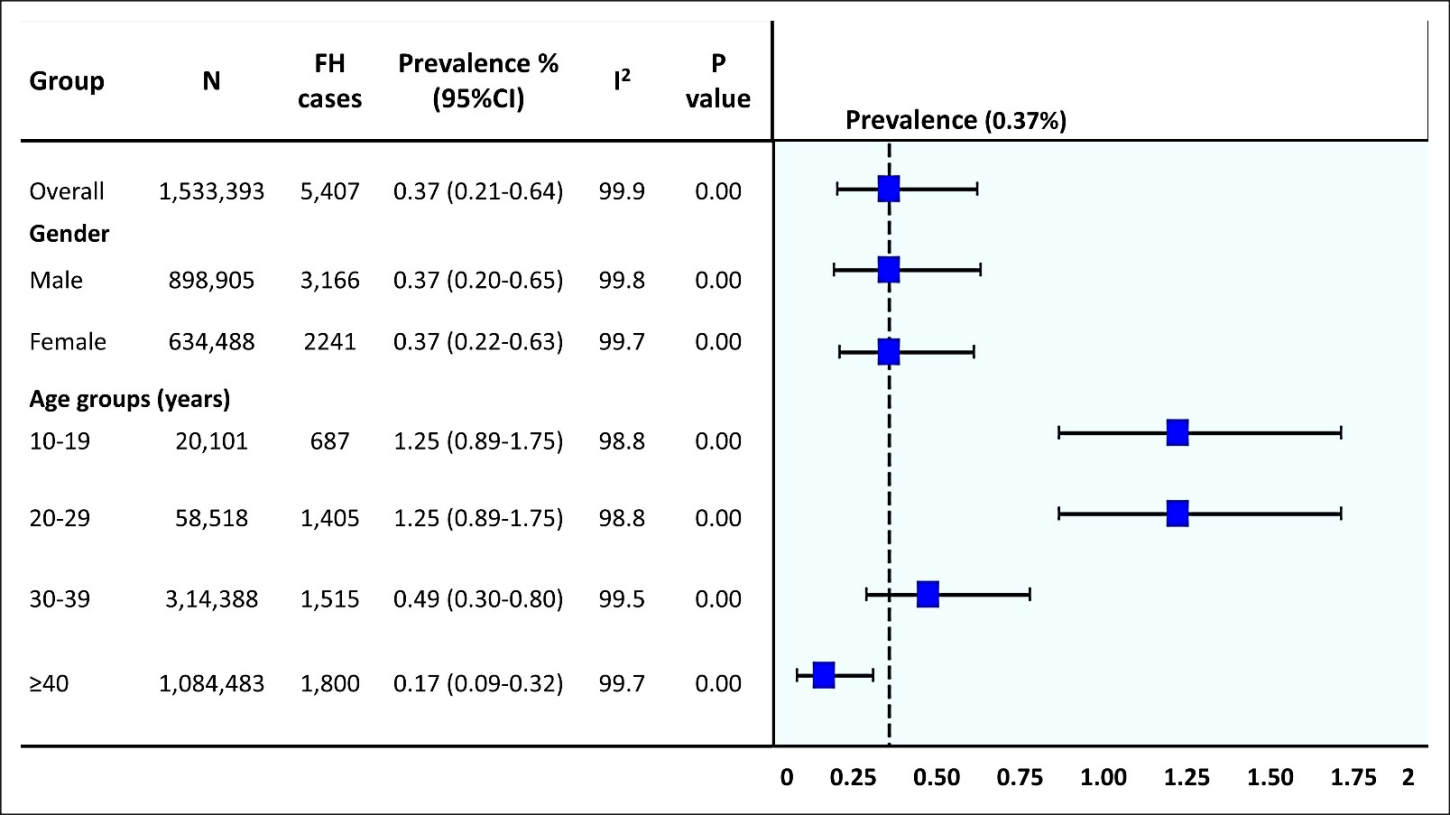
**Prevalence of FH in Pakistan in different groups based on age and gender**. P value is for I^2^.

Only one study was found that measured the prevalence of FH in Pakistan (11). Pooled prevalence estimates were calculated for the present study and the one identified. Both studies employed LDL-C MEDPED criteria for the characterization of the individuals. The pooled prevalence of FH in the population, derived from two studies from Pakistan encompassing 1,533,393 subjects (5,407 with FH), was estimated at 0.37% (95% CI = 0.21–0.64%) that is equivalent to around 1:273 individuals (Figure 2).

Stratified analysis based on age and gender revealed that the prevalence decreased with increasing age, where the prevalence was higher in individuals aged 10–19 years [1.25% (95% CI = 0.89–1.75%), 1:29) followed by 20–29 years (1.25% (95% CI = 0.89–1.75%), 1:34], 30–39 years [0.49% (95% CI = 0.30–0.80%), 1:221), and above 40 years [0.17% (95% CI = 0.09–0.32%), 1:573). There were no differences in prevalence of FH among males [0.37% (95% CI = 0.20–0.65%), 1:273] and females [0.37 (95% CI = 0.22–0.63%), 1:273] (Figure 2).

#### Prevalence of FH in South and Southeast Asia

**Figure 3:**
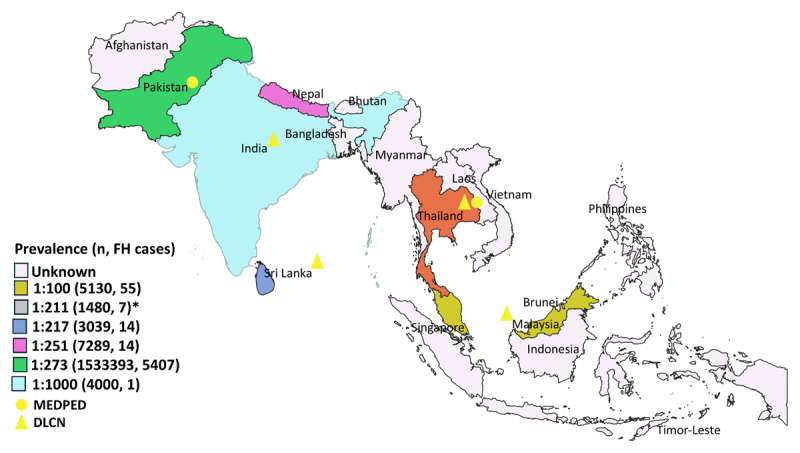
**Map of South and Southeast Asian countries showing the prevalence of FH**.

For most of the countries of South and Southeast Asia, no studies were found that had assessed the prevalence of FH (Figure 3). Studies reporting FH prevalence were from Pakistan, India, Sri Lanka, Malaysia, Singapore, Thailand (South region), and Nepal (Figure 3). Most of the studies utilized the Dutch Lipid Clinic Network (DLCN) criteria, while other utilized MEDPED, Simon Broome and American Heart Association (AHA) clinical criteria. The estimated prevalence of FH for different countries and the diagnostic criteria used were Malaysia (1:100, DLCN), Sri Lanka (1:217, DLCN), Nepal (1:251, DLCN, Simon Broome, AHA), Pakistan (1:273, MEDPED), and India (1:4000, DLCN) (13,14,15,16). In Thailand one study was conducted in the South region estimating prevalence of 1:211 individuals based on DLCN criteria (17).

## Discussion

The present study provides a comprehensive estimate of FH prevalence in Pakistan, utilizing an extensive dataset from two major laboratory networks. The analysis encompasses data from over 0.5 million individuals, representing all regions of Pakistan, with the highest representation from Punjab province. This approach builds upon and expands the previous study conducted in Pakistan, which utilized data from a single large diagnostic laboratory network, primarily representing the Sindh province (11). To estimate the prevalence of FH in Pakistan, we calculated a pooled prevalence by combining our results with those of the previous study. This meta-analysis, encompassing a total of 1,533,393 individuals, revealed an FH prevalence of 1:273 in Pakistan. Notably, this prevalence is slightly higher than the estimated global prevalence of 1:313 (6).

While considering subgroups, younger population, <20 years had the highest prevalence in both primary data (1:21) and pooled data (1:29). This high prevalence in the younger population could be due to selection bias, as it is not known whether testing was carried out for screening purposes or due to personal or family history of cardiovascular events in these individuals. A few other studies have also reported higher prevalence of FH in younger populations (6,11,18). No difference in prevalence among males and females were observed for pooled estimate (1:273) as previously observed (19,20). Other studies have reported contrasting trends of FH prevalence among genders (11,18), however, the number of males tested were higher compared to females which has been a consistent trend in several studies conducted in this region (11,21). Sociocultural factors play an important role in these differences where women tend to not seek healthcare (22).

The prevalence estimates from primary data suggest that the highest number of tests and subsequently cases were reported from the Punjab province, which is the largest province of Pakistan in terms of population. Despite being the largest province by area, the collection locations and cases were least in Balochistan, pointing toward an inequity in healthcare resource access among different regions of Pakistan (23). The pooled prevalence from both studies showed a higher number of cases from the province of Punjab and Sindh, however in both studies, the number of tests were also high for these two provinces.

In Pakistan, population-based estimates might be challenging due to lack of resources to conduct such studies. Despite no universal and agreed diagnostic criteria, genetic testing is recommended for accurate diagnosis and treatment (24). Since genetic testing is rarely conducted in Pakistan, utilizing lipid data to stratify the population based on their risk categories and conducting genetic testing in high-risk population can be a useful approach (10).

In more than 58% countries of South and Southeast Asia, the estimates on prevalence of FH in general population were not known, similar to the results of global study on FH prevalence where prevalence estimates were not known for about 90% of the countries (6). In most of the studies the prevalence of FH was estimated based on laboratory data suggesting a need for comprehensive population-based studies in the region. A few countries including Singapore and Nepal have already launched community-based screening programs (13,14).

There are a few limitations of this study. Since the data was derived from diagnostic laboratory, which may have introduced selection bias in the results and the samples might not represent actual population estimates. However, in low resource setting utilizing health record data can be the best alternative for making prevalence estimates. Moreover, due to the nature of data, details about comorbidities, family history, medications details are missing which might have effect on the overall prevalence estimates and estimating a low prevalence.

## Conclusion

This comprehensive study provides an updated estimate of FH prevalence in Pakistan and highlights the scarcity of data across South and Southeast Asia. Our findings indicate a higher FH prevalence in Pakistan than previously reported. There are significant gaps in FH epidemiological data for most countries in the region, suggesting the critical need for improved FH screening programs, especially targeting younger populations, and the development of region-specific management strategies. Large scale population-based studies to determine the burden of FH in South and Southeast Asia are needed that could be helpful in informing public health policies and resource allocation for early detection and treatment of FH.

## Data Accessibility Statement

The data underlying this article cannot be shared publicly due to ethical restrictions.

## Additional File

The additional file for this article can be found as follows:

10.5334/gh.1413.s1 Supplementary Material. This includes systematic review search strategy, along with additional tables and figures supporting the main findings.

## References

[1] Abifadel M, Boileau C. Genetic and molecular architecture of familial hypercholesterolemia. Journal of Internal Medicine. 2023;293(2):144–65. DOI: 10.1111/joim.13577 36196022 PMC10092380

[2] Vos T, Lim SS, Abbafati C, Abbas KM, Abbasi-Kangevari M, Abd-Allah F, et al. Global burden of 369 diseases and injuries in 204 countries and territories, 1990–2019: A systematic analysis for the Global Burden of Disease Study 2019. The Lancet. 2020;396(10258):1204–22. DOI: 10.1016/S0140-6736(20)30925-9 PMC7567026 33069326

[3] Migliara G, Baccolini V, Rosso A, D’Andrea E, Massimi A, Villari P, et al. Familial hypercholesterolemia: A systematic review of guidelines on genetic testing and patient management. Frontiers in Public Health. 2017;5:252. DOI: 10.3389/fpubh.2017.00252 28993804 PMC5622145

[4] Familial hypercholesterolaemia (FH): Report of a second WHO consultation, Geneva, 4 September 1998. World Health Organization; 1999. Available from: https://iris.who.int/handle/10665/66346

[5] Nordestgaard BG, Chapman MJ, Humphries SE, Ginsberg HN, Masana L, Descamps OS, et al. Familial hypercholesterolaemia is underdiagnosed and undertreated in the general population: Guidance for clinicians to prevent coronary heart disease. European Heart Journal. 2013;34(45):3478–90. DOI: 10.1093/eurheartj/eht273 23956253 PMC3844152

[6] Beheshti SO, Madsen CM, Varbo A, Nordestgaard BG. Worldwide prevalence of familial hypercholesterolemia: Meta-analyses of 11 million subjects. Journal of the American College of Cardiology. 2020;75(20):2553–66. DOI: 10.1016/j.jacc.2020.03.057 32439005

[7] Toft-Nielsen F, Emanuelsson F, Benn M. Familial hypercholesterolemia prevalence among ethnicities—Systematic review and meta-analysis. Frontiers in Genetics. 2022;13:840797. DOI: 10.3389/fgene.2022.840797 35186049 PMC8850281

[8] Bucholz EM, Rodday AM, Kolor K, Khoury MJ, De Ferranti SD. Prevalence and predictors of cholesterol screening, awareness, and statin treatment among US adults with familial hypercholesterolemia or other forms of severe dyslipidemia (1999–2014). Circulation. 2018;137(21):2218–30. DOI: 10.1161/CIRCULATIONAHA.117.032321 29581125 PMC6381601

[9] Raal FJ, Hovingh GK, Catapano AL. Familial hypercholesterolemia treatments: Guidelines and new therapies. Atherosclerosis. 2018;277:483–92. DOI: 10.1016/j.atherosclerosis.2018.06.859 30270089

[10] Sadiq F, Shafi S, Sikonja J, Khan M, Ain Q, Khan MI, et al. Mapping of familial hypercholesterolemia and dyslipidemias basic management infrastructure in Pakistan: A cross-sectional study. The Lancet Regional Health – Southeast Asia. 2023;100163. DOI: 10.1016/j.lansea.2023.100163 37384054 PMC10306043

[11] Farhad A, Noorali AA, Tajuddin S, Khan SD, Ali M, Chunara R, et al. Prevalence of familial hypercholesterolemia in a country-wide laboratory network in Pakistan: 10-year data from 988, 306 patients. Progress in Cardiovascular Diseases. 2023;79:19–27. DOI: 10.1016/j.pcad.2023.07.007 37516262

[12] Williams RR, Hunt SC, Schumacher MC, Hegele RA, Leppert MF, Ludwig EH, et al. Diagnosing heterozygous familial hypercholesterolemia using new practical criteria validated by molecular genetics. American Journal of Cardiology. 1993;72(2):171–6. DOI: 10.1016/0002-9149(93)90155-6 8328379

[13] Matthias AT, Samaranayake TSP, Hewa PS. Screening for familial hypercholesterolemia in Sri Lanka: A laboratory-based multicenter study. International Journal of Noncommunicable Diseases. 2024;9(2):58–64. DOI: 10.4103/jncd.jncd_100_23

[14] Sharma SK, Adhikari S, Shah N, Aebischer Perone S, Lab B, Heller O, et al. Familial hypercholesterolemia in community-based KHDC Nepal program-baseline data. European Journal of Preventive Cardiology. 2022;29(Supplement 1):265. DOI: 10.1093/eurjpc/zwac056.184

[15] Barde AK, Sethi S, Bhargav M, Waghdhare S. Indian prevalence of familial hypercholesterolemia demystified by applying Dutch lipid clinic network criteria. International Journal of Advances in Medicine. 2022;9(12):1177–82. DOI: 10.18203/2349-3933.ijam20223018

[16] Chua YA, Razman AZ, Ramli AS, Mohd Kasim NA, Nawawi H. Familial hypercholesterolaemia in the Malaysian community: Prevalence, under-detection and under-treatment. Journal of Atherosclerosis and Thrombosis. 2021;28(10):1095–107. DOI: 10.5551/jat.57026 33455995 PMC8560842

[17] Jeenduang N, Nuinoon M, Ratanawan C. Prevalence of familial hypercholesterolemia among the southern Thai population: A preliminary study; 2022. DOI: 10.21203/rs.3.rs-1046441/v1

[18] Jackson CL, Keeton JZ, Eason SJ, Ahmad ZA, Ayers CR, Gore MO, et al. Identifying familial hypercholesterolemia using a blood donor screening program with more than 1 million volunteer donors. JAMA Cardiolology. 2019;4(7):685–9. DOI: 10.1001/jamacardio.2019.1518 PMC6537793 31116347

[19] De Ferranti SD, Rodday AM, Mendelson MM, Wong JB, Leslie LK, Sheldrick RC. Prevalence of familial hypercholesterolemia in the 1999 to 2012 United States national health and nutrition examination surveys (NHANES). Circulation. 2016;133(11):1067–72. DOI: 10.1161/CIRCULATIONAHA.115.018791 26976914

[20] Klevmoen M, Mulder JWCM, Roeters van Lennep JE, Holven KB. Sex differences in familial hypercholesterolemia. Curr Atheroscler Rep. 2023;25(11):861–868. DOI: 10.1007/s11883-023-01155-6 37815650 PMC10618303

[21] Gupta R, Sharma M, Goyal NK, Bansal P, Lodha S, Sharma K. Gender differences in 7 years trends in cholesterol lipoproteins and lipids in India: Insights from a hospital database. Indian Journal of Endocrinology and Metabolism. 2016;20(2):211–8. DOI: 10.4103/2230-8210.176362 27042418 PMC4792023

[22] Roeters van Lennep JE, Tokgözoğlu LS, Badimon L, Dumanski SM, Gulati M, Hess CN, et al. Women, lipids, and atherosclerotic cardiovascular disease: A call to action from the European Atherosclerosis Society. European Heart Journal. 2023;44(39):4157–4173. DOI: 10.1093/eurheartj/ehad472 37611089 PMC10576616

[23] Farooq M, Usman M. Pakistan needs an equitable investment in the health system and collaborative efforts. Lancet Global Health. 2023;11(2):e177–8. DOI: 10.1016/S2214-109X(22)00523-X 36669797

[24] Cuchel M, Bruckert E, Ginsberg HN, Raal FJ, Santos RD, Hegele RA, et al. Homozygous familial hypercholesterolaemia: New insights and guidance for clinicians to improve detection and clinical management. A position paper from the Consensus Panel on Familial Hypercholesterolaemia of the European Atherosclerosis Society. European Heart Journal. 2014;35(32):2146–57. DOI: 10.1093/eurheartj/ehu274 25053660 PMC4139706

