# Supplementary material for: Prevalence of Familial Hypercholesterolemia in Pakistan: A Pooled Analysis of 1.5 Million Individuals and Comparison with Other Countries of the Region
Source: Glob Heart. 2025 Mar 10;20(1):23. doi: 10.5334/gh.1413 (PMC11908433; doi:10.5334/gh.1413)
Supplement: Supplementary Material. — This includes systematic review search strategy, along with additional tables and figures supporting the main findings. [file gh-20-1-1413-s1.pdf]

Supplementary material

Prevalence of Familial Hypercholesterolemia in Pakistan: A Pooled Analysis of 1.5 Million Individuals and Comparison with other Countries of the Region

Madeeha Khan, MS[1,2], Quratul Ain, MS[2,3], Jaka Sikonja, MD[4,5], Hijab Batool, FCPS[6], Muhammad Qasim Hayat, PhD[1], Mohammad Iqbal Khan, FRCS[7], Urh Groselj, MD,PhD[5,6**], Fouzia Sadiq, PhD[2*]

Madeeha Khan[1,2], Quratul Ain[2,3], Jaka Sikonja[4,5], Hijab Batool[6], Muhammad Qasim Hayat[1], Mohammad Iqbal Khan[7], Urh Groselj[5,6**], Fouzia Sadiq[2*]

[1]Atta ur Rehman School of Applied Biosciences, National University of Sciences and Technology, Islamabad
[2]Directorate of Research, Shifa Tameer-e-Millat University, Islamabad, Pakistan
[3]Translational Genomics Laboratory, Department of Biosciences, COMSATS University Islamabad, Pakistan
[4]Department of Endocrinology, Diabetes, and Metabolic Diseases, University Children's Hospital, University Medical Centre Ljubljana, Bohoriceva ulica 20, Ljubljana, Slovenia
[5]Faculty of Medicine, University of Ljubljana, Vrazov trg 2, Ljubljana, Slovenia
[6]Chemical Pathology, Chughtai Institute of Pathology, Lahore, Pakistan
[7]Department of Vascular Surgery, Shifa Tameer-e-Millat University, Shifa International Hospital Islamabad, Pakistan

1.1. Systematic review search strategy

"Familial hypercholesterolemia" OR "FH") AND (screening OR prevalence OR incidence OR epidemiology OR frequency)) AND ("South Asia" OR "Southeast Asia" OR India OR Pakistan OR Bangladesh OR "Sri Lanka" OR Nepal OR Bhutan OR Maldives OR Afghanistan OR Indonesia OR Malaysia OR Singapore OR Philippines OR Thailand OR Vietnam OR Myanmar OR Burma OR Cambodia OR Laos OR Brunei OR "Timor-Leste"

1.2. Supplementary figures

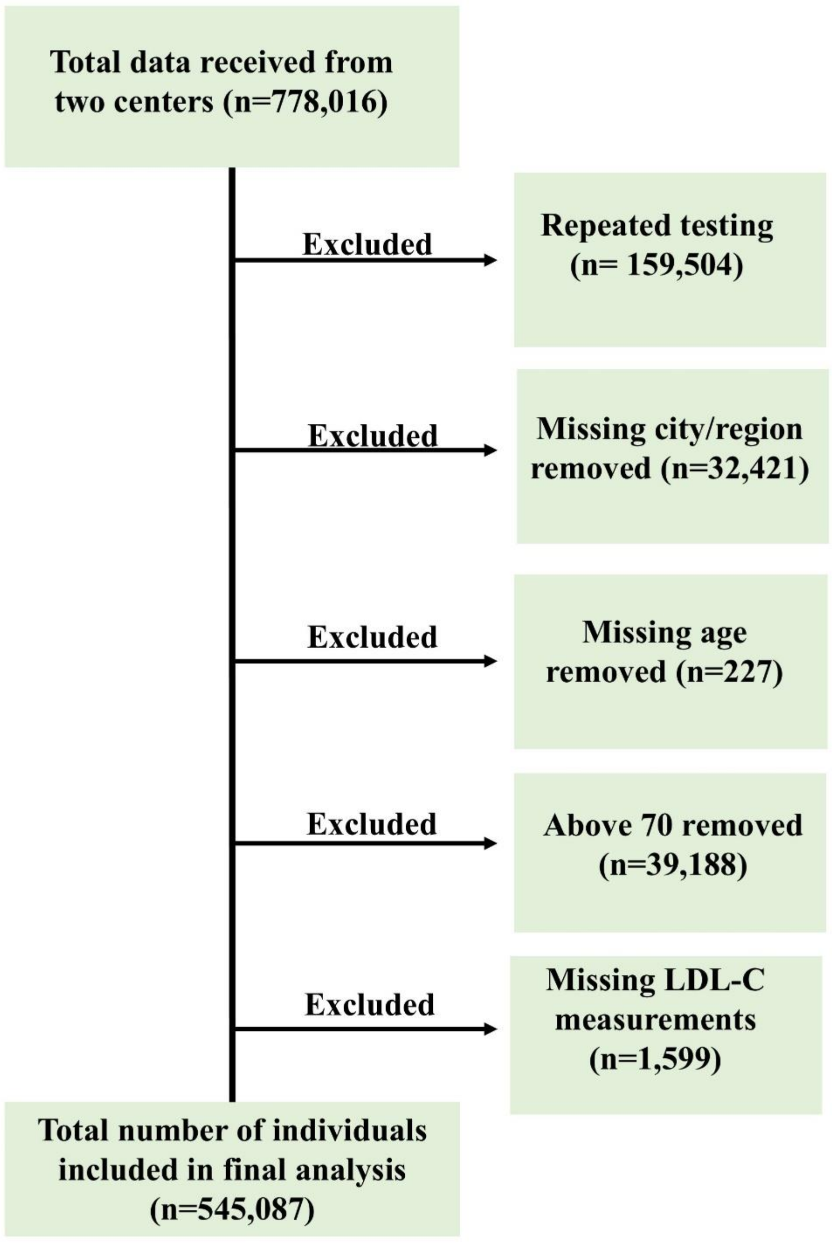

Supplementary Figure 1: Methodological framework for inclusion of participants of the study

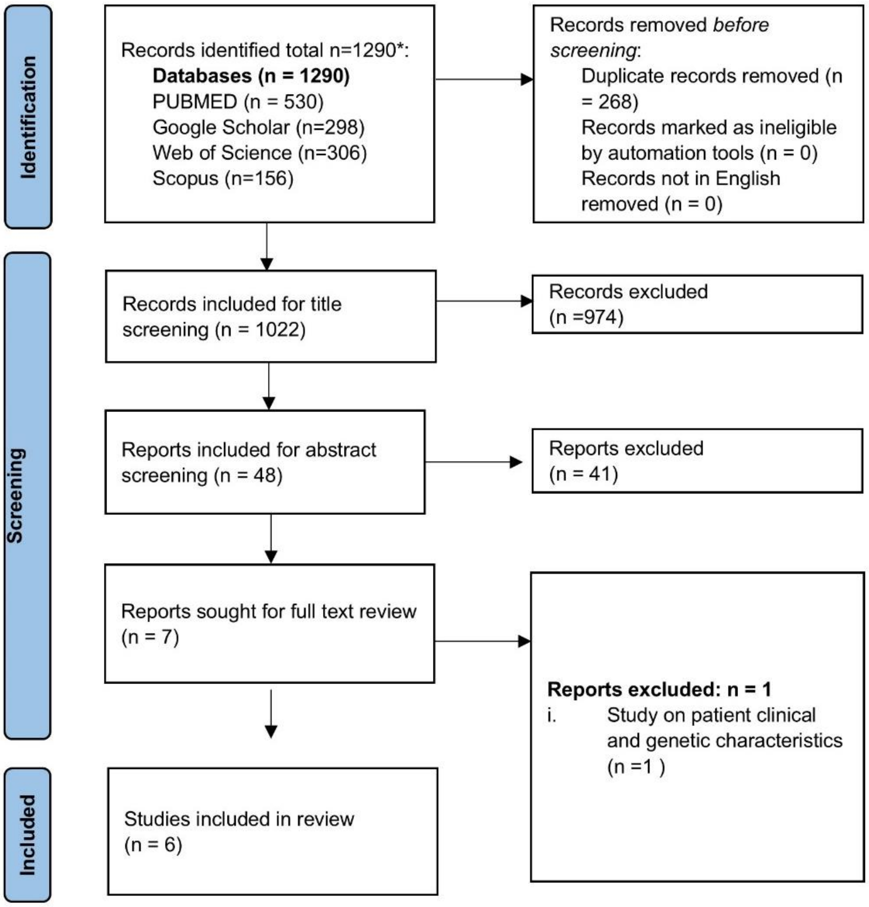

*Consider, if feasible to do so, reporting the number of records identified from each database or register searched (rather than the total number across all databases/registers).

From: Page MJ, McKenzie JE, Bossuyt PM, Boutron I, Hoffmann TC, Mulrow CD, et al. The PRISMA 2020 statement: an updated guideline for reporting systematic reviews. BMJ 2021;372:n71. doi: 10.1136/bmj.n71

Supplementary Figure 2: PRISMA flowchart for the systematic review of literature

Supplementary Table 2: Characteristics and quality assessment scores of the studies included in the systematic review

Sr no	Study	Authors	Publication year	Country	Setting	Sample size (n)	FH cases	FH diagnostic criteria	Study quality* (Score)	Reference
1	Familial Hypercholesterolaemia in the Malaysian Community: Prevalence, Under-Detection and Under-Treatment	Chua YA, Razman AZ, Ramli AS, Mohd Kasim NA, Nawawi H.	2021	Malaysia	General population	5130	55	DLCN	High (8)	(1)
2	Indian prevalence of Familial Hypercholesterolemia demystified by applying Dutch lipid clinic network criteria	Barde, Akshay Kacharu; Sethi, Sumit; Bhargav, Mohan; Waghdhare, Swati;	2022	India	Single centre	4000	4	DLCN	High (7)	(2)
3	Familial Hypercholesterolemia in community-based KHDC Nepal program-baseline data	Sharma, SK; Adhikari, S; Shah, N; Aebischer Perone, S; Lab, B; Heller, O; Chappuis, F;	2022	Nepal	General population	7289	29	DLCN, Simon Broome, AHA clinical criteria	Moderate (6)	(3)
4	Prevalence of Familial Hypercholesterolemia in a country-wide laboratory network in Pakistan: 10-year data from 988, 306 patients	Farhad A, Noorali AA, Tajuddin S, Khan SD, Ali M, Chunara R, Khan AH, Zafar A, Merchant A, Bokhari SS,	2023	Pakistan	Laboratory network	988306	2416	MEDPED	High (8)	(4)

		Virani SS, Samad Z.								
5	Screening for Familial Hypercholesterolemia in Sri Lanka: A laboratory-based multicenter study	Matthias, Anne Thushara; Samaranayake, TSP; Hewa, Peduru Saman;	Sri Lanka	2024	Laboratory network	3039	14	DLCN	High (8)	(5)
6	Prevalence of Familial Hypercholesterolemia among the southern Thai population: A preliminary study	Jeenduang, Nutjaree; Nuinoon, Manit; Ratanawan, Chutima;	Thailand	2022	General population	1480	7	DLCN	Moderate (6)	(6)
*Quality assessment based on Joanna Briggs Institute (JBI) Prevalence Critical Appraisal Tool, DLCN: Dutch lipid clinic network criteria, MEDPED: Make Early Diagnosis to Prevent Early Death, AHA: American Heart Association										

Supplementary references

1. Chua YA, Razman AZ, Ramli AS, Mohd Kasim NA, Nawawi H. Familial Hypercholesterolaemia in the Malaysian Community: Prevalence, Under-Detection and Under-Treatment. *Journal of Atherosclerosis and Thrombosis. 2021*;28(10):1095–107. doi: 10.5551/jat.57026

2. Barde AK, Sethi S, Bhargav M, Waghdhare S. Indian prevalence of familial hypercholesterolemia demystified by applying Dutch lipid clinic network criteria. *International Journal of Advances in Medicine*. 2022;9(12):1177–82. 10.18203/2349-3933.ijam20223018

3. Sharma SK, Adhikari S, Shah N, Aebischer Perone S, Lab B, Heller O, et al. Familial hypercholesterolemia in community-based KHDC Nepal program-baseline data. *European Journal of Preventive Cardiology*. 2022;29(Supplement_1):265. doi: 10.1093/eurjpc/zwac056.184

4. Farhad A, Noorali AA, Tajuddin S, Khan SD, Ali M, Chunara R, et al. Prevalence of familial hypercholesterolemia in a country-wide laboratory network in Pakistan: 10-year data from 988, 306 patients. *Progress in Cardiovascular Diseases*. 2023; 79:19-27. doi: 10.1016/j.pcad.2023.07.007

5. Matthias AT, Samaranayake TSP, Hewa PS. Screening for familial hypercholesterolemia in Sri Lanka: A laboratory-based multicenter study. *International Journal of Noncommunicable Diseases*. 2024;9(2):58–64. doi: 10.4103/jncd.jncd_100_23

6. Jeenduang N, Nuinoon M, Ratanawan C. Prevalence of familial hypercholesterolemia among the southern Thai population: A preliminary study. 2022; doi: 10.21203/rs.3.rs-1046441/v1
